# Ripoptocide – A Spark for Inflammation

**DOI:** 10.3389/fcell.2019.00163

**Published:** 2019-08-13

**Authors:** Rosalind L. Ang, Mark Chan, Adrian T. Ting

**Affiliations:** ^1^Precision Immunology Institute, Icahn School of Medicine at Mount Sinai, New York, NY, United States; ^2^MSBS Program, Graduate School of Biomedical Sciences, Icahn School of Medicine at Mount Sinai, New York, NY, United States

**Keywords:** TNF, ripoptocide, apoptosis, necroptosis, ubiquitin, E3 ligase, deubiquitinase, RIPK1

## Abstract

The clinical success of biologics that inhibit TNF (Tumor Necrosis Factor) in inflammatory bowel diseases (IBD), psoriasis and rheumatoid arthritis (RA) has clearly established a pathogenic role for this cytokine in these inflammatory disorders. TNF binding to its receptors activates NFκB and MAPK signaling, inducing the expression of downstream pro-inflammatory genes. This is thought to be the primary mechanism by which TNF elicits inflammation. TNF is also a well-known trigger of caspase-dependent apoptosis or caspase-independent necroptosis. Whether cell death has any role in TNF-mediated inflammation has been less clear. Emerging data from animal models now suggest that cellular demise caused by TNF may indeed provoke inflammation. The default response of most cells to TNF stimulation is survival, rather than death, due to the presence of two sequential cell death checkpoints. The early checkpoint is transcription-independent involving the non-degradative ubiquitination of RIPK1 to prevent RIPK1 from becoming a death-signaling molecule. The later checkpoint requires the induction of pro-survival genes by NFκB-mediated transcription. When the early checkpoint is disrupted, RIPK1 initiates cell death and we suggest the term *ripoptocide* to describe this manner of death (encompassing both apoptosis and necroptosis). The sensitivity of a cell to ripoptocide is determined by the balance between regulatory molecules that enforce and those that disassemble the early checkpoint. As there is evidence suggesting that ripoptocide is inflammatory, individuals may develop inflammation due to ripoptocide brought about by genetic, epigenetic or post-translational alteration of these checkpoint regulators. For these individuals, drugs that reinforce the early checkpoint and inhibit ripoptocide could be useful in ameliorating inflammation.

## Role of TNF in Inflammation

Tumor necrosis factor (TNF) was first described in 1975 as a serum factor that could lyse tumor cells present in bacillus Calmette-Guerin (BCG)-infected mice that were challenged with endotoxin (Carswell et al., 1975). It was discovered as part of an effort to uncover factors that could account for the observations of William Coley in the late 1800s, who administered a bacterial cocktail to induce tumor regression in his patients. This bacterial cocktail came to be known as Coley’s toxins. Subsequent efforts to use TNF as an anti-tumor agent in patients largely failed, due in part to the fact that TNF administration induces an intolerable systemic inflammatory response. TNF is now used only in conjunction with melphalan in isolated limb perfusion (TM-ILP) for the localized treatment of soft tissue sarcoma and melanoma of the extremities (Grunhagen et al., 2006). A large body of work over the past few decades has now shown TNF to be highly inflammatory with pleiotropic effects in multiple cells and tissues. This pro-inflammatory function of TNF plays a physiological role in anti-microbial defense (Fiers, 1991). On the other hand, dysregulation of TNF has been linked to the development of inflammatory diseases including rheumatoid arthritis (RA), inflammatory bowel diseases (IBD) and psoriasis. Biologics that block TNF have proven to be highly effective in the treatment of these inflammatory disorders (Taylor and Feldmann, 2009; Blandizzi et al., 2014; Mitoma et al., 2018). This inflammatory role of TNF in both anti-microbial defense and in inflammatory disorders is thought to be due to its induction of NFκB and MAPK signaling, and subsequent transcription of downstream pro-inflammatory genes including other cytokines, chemokines, receptors and adhesion molecules.

Another explanation for why TNF failed as an anti-tumor agent is that TNF is a poor inducer of tumor cell death when used as a single agent, contrary to its initial description as a cytotoxic factor. The initial experiments were carried out using Meth A and L929 mouse tumor lines (Carswell et al., 1975), which are highly sensitive to TNF-induced cell death. In contrast, most transformed cells as well as non-transformed primary cells are largely resistant to TNF-induced cytotoxicity. Indeed, TNF has the opposite effect and induces a pro-survival state in most cells. Nonetheless, extensive studies have demonstrated that TNF has the capability to induce cell death under the right circumstances. Experimentally, this often involved the use of pharmacological agents or genetic manipulation to sensitize cells to death. One manipulation used often to sensitize cells to TNF-induced killing is to treat cells with either actinomycin D or cycloheximide to block new protein synthesis. This indicated that the cell death machinery is pre-existing but since the default response to TNF in most cells is survival, this suggested that there are molecular mechanisms that serve as checkpoints to suppress the cell death machinery. Since the default response is survival rather than death, the physiological and patho-physiological function of TNF-induced cytotoxicity has been difficult to study. While knocking out TNF enables one to ascribe a role for TNF to a particular biological response, one is unable to conclude whether that TNF-mediated response is due to its induction of MAPK/NFκB signaling or cell death. Therefore, a role for cell death in mediating the inflammatory effects of TNF has been unclear. Recent emerging data from mouse genetic models with perturbations that alters the cell death response now support the notion that cell death may play a role in driving inflammatory responses. In this review, we will discuss our current understanding of the molecular mechanisms that determine whether a cell remains resistant or succumb to TNF-induced death and propose that tipping the response to death may be linked to inflammation in some patients.

## Dual Sequential Cell Death Checkpoints in the TNF Pathway

**FIGURE 1:**
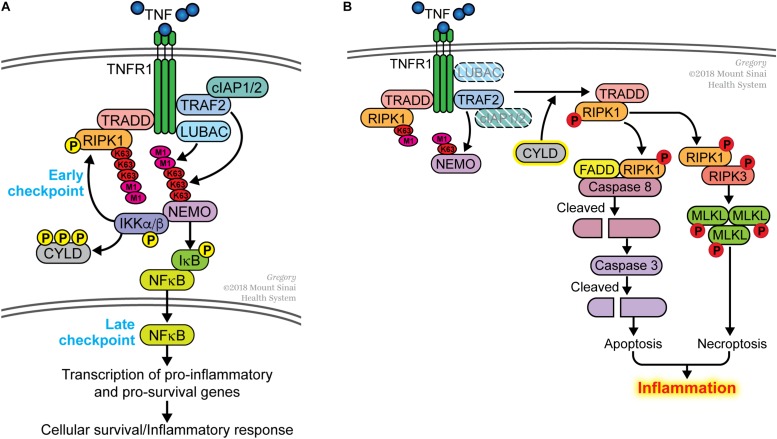
Dual sequential cell death checkpoints in the TNFR1 signaling pathway. **(A)** In most cells, binding of TNF to its receptor TNFR1 does not trigger cell death. This is due to the presence of two sequential cell death checkpoints. The early checkpoint occurs rapidly following TNFR1 ligation when RIPK1 is conjugated with K63- and M1-linked poly-ubiquitin chains catalyzed by the TRAF2/cIAP1/2 and LUBAC E3 ligases, respectively. When this occurs, RIPK1 functions as pro-survival signaling molecule and does not associate with the FADD/CASPASE 8 death-signaling complex. This early checkpoint is reinforced by IKK-mediated phosphorylation of RIPK1 and CYLD to further inhibit their death-signaling capabilities. The late checkpoint occurs when NFκB translocates to the nucleus to upregulate expression of pro-survival genes. The two checkpoints function in different ways to protect cells from death; the early checkpoint does not require *de novo* gene transcription whereas the later checkpoint does. **(B)** The early checkpoint fails when RIPK1 ubiquitination is disrupted. This happens when the ubiquitin E3 ligases (e.g., TRAF2, cIAP1/2, LUBAC) is inhibited or deleted. An increase in the activity of deubiquitinases (e.g., CYLD), which remove ubiquitin from RIPK1, can similarly lead to the disruption of the early checkpoint. Impaired ubiquitination of RIPK1 leads to its association with FADD and CASPASE 8 to initiate apoptosis, or with RIPK3 to induce necroptosis if apoptosis is inhibited. We use the term ripoptocide to denote this RIPK1-dependent death, which can lead to inflammation.

The early observation that transcription or translation inhibitors sensitized cells to TNF-induced death pointed toward the presence of a transcription-dependent cell death checkpoint. In the mid-1990s, this checkpoint was attributed to NFκB-dependent transcription of pro-survival genes (Beg and Baltimore, 1996; Van Antwerp et al., 1996; Wang et al., 1996). One critical molecule induced by NFκB is c-FLIP, which binds to unprocessed CASPASE 8 and prevents it from triggering apoptosis (Micheau et al., 2001; Micheau and Tschopp, 2003). c-FLIP is a short-lived protein and if it is not replenished by NFκB-dependent transcription, unprocessed CASPASE 8 undergoes autocatalysis to generate a p18/p10 tetrameric complex that initiates the apoptotic cascade. Other pro-survival molecules induced by NFκB includes members of the BCL2 family and several components of the TNF receptor 1 (TNFR1) signaling complex such as cIAP1/2, TRAF2 and A20 (Wang et al., 1998, 1999; Lee et al., 2000; He and Ting, 2002). Another checkpoint was discovered in 2007 and this was shown to be dependent on the non-degradative ubiquitination of the TNF signaling molecule RIPK1 but did not depend on NFκB-mediated transcription (O’Donnell et al., 2007). We had previously proposed that these two cell death checkpoints function sequentially in the TNFR1 signaling pathway (O’Donnell and Ting, 2010, 2011; Ting and Bertrand, 2016). Ubiquitination of RIPK1 functions as the initial checkpoint and this transcription-independent checkpoint serves to prevent RIPK1 from becoming a survival-signaling molecule (Figure 1A). The TRAF2/cIAP1/2 and LUBAC E3 ubiquitin ligases are recruited to TNFR1 to conjugate K63-linked and M1-linked (linear) polyubiquitin chains onto RIPK1, respectively (Hsu et al., 1996; Shu et al., 1996; Bertrand et al., 2008; Wang et al., 2008; Haas et al., 2009; Gerlach et al., 2011). Polyubiquitinated RIPK1 serves as a platform to recruit the TAB2/3-TAK1 and NEMO-IKKα/β kinase complexes allowing TAK1 to phosphorylate and activate IKKα/β (Figure 1A). One function of NEMO-IKKα/β is to phosphorylate RIPK1 on residue serine 25 to further suppress its death-signaling capability (Dondelinger et al., 2015, 2019), as well as CYLD to inhibit this deubiquitinase from dismantling K63-linked polyubiquitin chains (Reiley et al., 2005). The death-signaling capability of RIPK1 is additionally suppressed by TAK1, TBK1/IKKε and MK2–mediated phosphorylation (Dondelinger et al., 2017; Geng et al., 2017; Jaco et al., 2017; Menon et al., 2017; Lafont et al., 2018; Xu et al., 2018). These phosphorylation events serve to reinforce the early checkpoint, providing a transient protection against death. Another function of NEMO-IKKα/β is to phosphorylate I-κBα leading eventually to the activation of NFκB and its induction of pro-survival genes (Figure 1A). This later checkpoint constitutes a transcription-dependent programing of the cells to provide a more permanent protection against death. Indeed, gene products of the late NFκB-dependent checkpoint include the E3 ligase for RIPK1 (i.e., cIAP1/2 and TRAF2) thereby functioning in a positive feedback manner to further strengthen the early checkpoint and to suppress RIPK1’s death-signaling function. A20/TNFAIP3 is another gene product induced by NFκB that strengthens the early checkpoint by binding to M1-linked ubiquitin chains and preventing their dismantling (Draber et al., 2015).

While disruption of either checkpoint sensitizes cells to TNF-induced death, the manner by which the cells die are different in the two situations. The early checkpoint can be disrupted by inhibiting ubiquitination of RIPK1. This can be done experimentally by treating cells with SMAC mimetics that degrade cIAP1/2 (Bertrand et al., 2008), mutating the ubiquitin acceptor site on RIPK1 (O’Donnell et al., 2007), deleting NEMO (Legarda-Addison et al., 2009) or the E3 ligases that catalyze K63-linked or M1-linked (linear) ubiquitin conjugation (Gerlach et al., 2011; Ikeda et al., 2011; Tokunaga et al., 2011; Moulin et al., 2012). Disrupting ubiquitination ‘flips on a death switch’ on RIPK1 (O’Donnell et al., 2007), converting it from a survival-signaling molecule to a death-signaling molecule. This enables RIPK1 to associate with the FADD/CASPASE 8 complex leading to the auto-processing of CASPASE 8 to initiate apoptosis (Figure 1B). If FADD/CASPASE 8 is absent or defective, RIPK1 forms a complex with RIPK3, leading to the activation of this kinase. RIPK3 phosphorylates MLKL and this initiates an alternative form of cell death known as necroptosis or programed necrosis. In either case, this can be considered ‘death by execution’ and a hallmark of this death is the requirement for a functional RIPK1 kinase activity. We propose the term *ripoptocide* to describe cell death that is dependent on RIPK1, be it apoptosis or necroptosis. Another route to flip on the death switch on RIPK1 is to activate CYLD, a deubiquitinase that preferentially dismantles K63-linked ubiquitin chains, including from RIPK1. On the other hand, the late checkpoint can be disrupted by pharmacological or genetic inhibition of NFκB-dependent gene expression. This leads to a failure in replenishing c-FLIP, which normally acts as a brake on CASPASE 8. Without c-FLIP present, CASPASE 8 undergoes auto-processing and initiates apoptosis (Micheau and Tschopp, 2003). This can be considered ‘death by starvation’ and RIPK1 is not involved in turning on this death. While TNF can induce apoptosis if either checkpoint is disrupted, because the mechanisms involved are different, it is likely that the biological effects of ‘death by execution’ and ‘death by starvation’ is likely to be different.

## Apoptosis Conferred by the Loss of the Early Checkpoint is Inflammatory

Apoptotic cell death has been largely assumed to be non-inflammatory and tolerogenic, and there are examples in the immune system that support this idea. For instance, the bulk of thymocytes undergo apoptosis when they failed to be selected (Wiegers et al., 2011; Daley et al., 2017) with no sign of inflammation in the thymus. Similarly, antigen-specific lymphocytes that multiply in response to an infection undergo apoptosis after the infection has been cleared due to cytokine withdrawal without causing inflammation (Snow et al., 2010). The rapid engulfment of apoptotic cellular debris by phagocytic cells to prevent the release of intracellular content, together with presentation of self-antigens on MHC molecules in a non-inflamed environment without costimulatory signals are ways by which apoptosis can induce peripheral tolerance (Ferguson et al., 2011; Martin et al., 2012; Green et al., 2016).

TNF can induce RIPK1/RIPK3-dependent necroptosis, a form of necrotic death marked by the release of endogenous ligands for pattern recognition receptors (known as damage-associated molecular patterns or DAMPs), which can activate innate immune cells to mount an inflammatory response (Wallach et al., 2016). Since existing paradigm considers apoptosis to be tolerogenic, TNF-induced necroptosis rather than apoptosis is thought to induce inflammation. However, emerging animal models are now suggesting that TNF-induced apoptosis can in fact be inflammatory as well. A key model that has shed light on this is the *cpdm* mutant mouse strain. This strain was identified because it spontaneously developed dermatitis and was subsequently shown to possess a loss-of-function mutation in the *Sharpin* gene. In addition to the skin, the *cpdm* strain also exhibits multi-organ inflammation and immunodeficiency (HogenEsch et al., 1999). SHARPIN associates with two RING-containing proteins, HOIP/RNF31 and HOIL1/RBCK1, to form the Linear Ubiquitination Assembly Complex (LUBAC) (Ikeda, 2015). LUBAC is an E3 ligase that catalyzes the addition of linear ubiquitin chains on RIPK1 and NEMO, and this post-translational modification is a critical part of the early checkpoint (Figure 1B). SHARPIN deficiency conferred sensitivity to RIPK1-dependent death in cells treated with TNF (Gerlach et al., 2011; Ikeda et al., 2011; Tokunaga et al., 2011). *In vivo*, the dermatitis seen in the *Sharpin*-deficient *cpdm* strain can be reversed by a compound deletion in *Tnf* (Gerlach et al., 2011) indicating that this inflammation is TNF-driven. Furthermore, a K45A knock-in mutation of *Ripk1* that disables its kinase activity (and thus RIPK1-dependent death) also reversed the skin inflammation in the *cpdm* mice (Berger et al., 2014). This observation demonstrated that the SHARPIN deficiency led to the disruption of the early checkpoint and flipped on the death switch on RIPK1. Conditional deletion of the death-signaling molecule *Fadd* in keratinocytes (Kumari et al., 2014) or a heterozygous germline deletion of *Casp8* (Rickard et al., 2014), both in combination with germline *Ripk3* deletion to disable apoptosis and necroptosis, also reversed the inflammation in the *cpdm* strain. However, deletion of just *Ripk3* or *Mlkl* in the *cpdm* strain only partially reversed the skin inflammation (Kumari et al., 2014; Rickard et al., 2014). The *Sharpin*^cpdm/cpdm^*Ripk3*^–/–^ or *Sharpin*^cpdm/cpdm^*Mlkl*^–/–^ mice, which were competent for TNF-induced apoptosis but not necroptosis, still developed dermatitis.

Another insightful model came from the study of mice with a deletion of *Nemo*, a component of the IKK complex, in intestinal epithelial cells (IEC). Prior *in vitro* studies had indicated that NEMO is an essential component of the early checkpoint (Legarda-Addison et al., 2009; O’Donnell et al., 2012). NEMO can function as a ubiquitin-dependent physical restraint on RIPK1 (Legarda-Addison et al., 2009; O’Donnell et al., 2012) or via IKK-dependent phosphorylation of RIPK1 (Dondelinger et al., 2015) to inhibit the death-signaling function of RIPK1. Since NEMO is also essential for NFκB signaling, loss of NEMO leads to the failure of both checkpoints but cell death in NEMO-deficient cells is dependent on RIPK1 (Legarda-Addison et al., 2009; O’Donnell et al., 2012), indicating that the early checkpoint is central to cell death sensitivity. IEC-specific deletion of *Nemo* led to severe intestinal inflammation that is TNF-dependent and reversed with a loss-of-function in the kinase domain of RIPK1 (Vlantis et al., 2016). In contrast, combined deletion of *Rel* members of the NFκB family in the same tissue (thereby leading to failure of only the late checkpoint with no RIPK1 involvement) did not lead to colitis (Vlantis et al., 2016). Similar to the situation in the *cpdm* mice, deletion of *Ripk3* in the IEC knockout of *Nemo*, still resulted in colitis in a proportion of mice (Vlantis et al., 2016). In both models, blocking RIPK3-dependent necroptosis did not completely reversed the inflammation. Another insightful model is the *Tnfaip3^–/–^* mice, which succumbed postnatally to multi-organ inflammation (Lee et al., 2000). This postnatal lethality can be partially reversed by inactivating the kinase activity of RIPK1 or by deleting *Ripk3* but not deleting *Mlkl* (Newton et al., 2016). Thus, the inflammation in A20-deficient mice is not due to MLKL-mediated necroptosis but rather, it is caused in part by RIPK1/3-dependent apoptosis or a death-independent function. It is interesting to note that since A20 is also an inhibitor of NFκB signaling, A20-deficient cells also harbor enhanced NFκB activity but despite this, A20-deficient cells are highly sensitive to TNF-induced apoptosis (Lee et al., 2000). This behavior suggests that disruption in the early checkpoint (and therefore ripoptocide) can override a functioning late checkpoint. It should also be noted that the postnatal lethality of the A20-deficient mice could not be fully reversed by the kinase-inactive RIPK1 or RIPK3 removal (Newton et al., 2016), suggesting that the inflammation caused by A20 deficiency may be due to a combination of excessive cell death and NFκB gene transcription. These different mouse knockout models suggest that TNF-driven apoptosis caused by disruption of the early checkpoint (and therefore dependent on RIPK1) underlies the inflammation. Thus, there may be something uniquely inflammatory about RIPK1-dependent apoptosis. One possibility is that in addition to activating the cell death machinery, RIPK1 can also induce the expression of inflammatory cytokines and chemokines in the dying cells (Yatim et al., 2015; Najjar et al., 2016; Saleh et al., 2017; Zhu et al., 2018). The combined effect of apoptosis with inflammatory cytokines/chemokines may be particularly potent at recruiting and activating inflammatory cells. Another possibility is that TNF-induced cell death is a combination of apoptosis and necroptosis in contexts where RIPK3 is available. RIPK1-dependent apoptosis could also lead to inflammation if this is occurring in cells serving a barrier function. Their inappropriate loss would lead to a breach in barrier and subsequent invasion of the underlying tissue by commensals.

The mouse models described above, which are caused by single gene alteration, provide evidence that disruption of the early checkpoint results in ripoptocide and inflammation. Humans with a genetic defect in the early checkpoint would be similarly expected to develop inflammation. In this regard, humans with a genetic defect in *RNF31* (coding for HOIP) or *RBCK1* (coding for HOIL1) developed autoinflammation and immunodeficiency, which overlap with the phenotype observed in the SHARPIN-deficient mice. Females with a single copy defect in the X-linked *IKBKG* gene (coding for NEMO) develop Incontinentia Pigmenti (IP), which is characterized by skin inflammation during the early stages of life (Fusco et al., 2015). Cells in which X-inactivation occurred on the wild type *IKBKG* allele would be sensitive to TNF-induced ripoptocide. Humans haploinsufficient for *TNFAIP3* (coding for A20) also developed autoinflammation (Zhou et al., 2016), akin to the phenotype of the *Tnfaip3*^–/–^ mice. In addition, polymorphisms in the *TNFAIP3* gene has long been associated with a number of human inflammatory disorders (Vereecke et al., 2011; Ma and Malynn, 2012). While there is no direct evidence currently that the inflammation in these human genetic disorders is RIPK1-dependent, the mouse models strongly suggest that the pathology in these genetic disorders is caused by a failure in the early checkpoint. Therapeutically, these rare patients may benefit from the use of TNF antagonists and RIPK1 kinase inhibitors.

## Mechanisms that Confer Sensitivity to Ripoptocide and Inflammation

While the genetic models described above provide insights into the biological consequence of disrupting the early checkpoint, it is less clear how this checkpoint may be disrupted in a normal individual. The TNF cell death pathway likely evolved as an anti-microbial defense mechanism (Old, 1985) and the physiological role of the early checkpoint in this response remains incomplete. Germline deletion of several components of the early checkpoint (e.g., cIAP1/2, HOIP, HOIL1 and TRAF2) resulted in embryonic lethality due to inappropriate cell death (Yeh et al., 1997; Moulin et al., 2012; Peltzer et al., 2014, 2018) demonstrating that these survival molecules are essential for development. However, this checkpoint and more importantly, the capability to actively induce death when it fails, must serve an essential postnatal function evolutionary because there is no selection pressure to have this death response in a developing embryo. The early checkpoint likely evolved to serve a ‘trapdoor’ function in postnatal life. The molecules that constitute the early checkpoint are often also involved in signaling downstream of pattern recognition and cytokine receptors. Thus, these checkpoint molecules are targeted by microbial-encoded effector molecules to block the pattern recognition and cytokine receptors from signaling (Silke and Hartland, 2013). In so doing, the infected cells become vulnerable to TNF-induced ripoptocide and this could serve to limit infection. The fact that microbes encode molecules that block apoptosis is consistent with the notion that the induced death of host cells serves an anti-microbial function (Silke and Hartland, 2013). In addition, the pro-inflammatory effects of ripoptocide could serve to bypass the inflammatory blockade imposed by the microbial-encoded molecules. There is evidence that an effective response to Yersinia infection requires RIPK1-dependent apoptosis (Peterson et al., 2017; Dondelinger et al., 2019). The Yersinia effector molecules YopJ can target components of the early checkpoint including TAK1 and IKK (Orning et al., 2018; Dondelinger et al., 2019). Recently, it was reported that TBK1 and IKKε phosphorylate RIPK1 to inhibit its death-signaling function (Lafont et al., 2018; Xu et al., 2018) and this constitutes another element of the early checkpoint. Since TBK1 and IKKε play a critical role in the induction of type I interferon, there is speculation that microbial-encoded antagonists of TBK1/IKKε, in attempting to block type I interferon induction, could open the ‘trapdoor’ leading to TNF-mediated destruction of infected cells.

**FIGURE 2:**
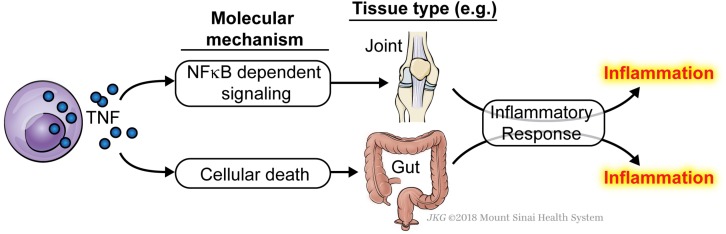
TNF can cause inflammation via induction of either NFκB signaling or ripoptocide. Induction of either arm of the signaling pathways downstream of TNFR1 can lead to inflammation. It may be that in some tissue, the inflammation is caused by excessive TNF-induced NFκB signaling and expression of downstream cytokines, adhesion molecules and other pro-inflammatory genes. In a different tissue, the inflammation may be caused by excessive TNF-induced ripoptocide, which could come about from reduced expression of signaling molecules that fortify the early checkpoint. The affected tissues are drawn for illustrative purposes. Currently, there is no evidence to show tissue-dependent sensitivity to ripoptocide. Alternatively, within a single tissue/disorder, some patients develop inflammation due to excessive NFκB signaling whereas others develop inflammation due to excessive ripoptocide.

Since TNF underlies a number of inflammatory disorders, the question arises as to whether the inflammation in these pathologies is caused by excessive TNF-driven NFκB and MAPK signaling, or by a failure in the early checkpoint leading to inappropriate TNF-induced ripoptocide. We propose that this may be dependent on the tissue affected and on the individual. For instance, it is possible that in one inflammatory disorder, it is caused by excessive TNF-driven expression of NFκB-dependent inflammatory genes whereas in a different disorder, it is driven by TNF-mediated death (Figure 2). It may be that in a particular tissue, the affected cell type expresses lower level of checkpoint-fortifying molecules rendering this cell more susceptible to ripoptocide. However, in tissues where early checkpoint molecules are highly expressed, inflammation may be due to excessive induction of NFκB and MAPK signaling. It is also possible that within a population of patients with the same disorder, some develop inflammation due to excessive cell death whereas others develop inflammation due to excessive NFκB/MAPK signaling. RIPK1 kinase inhibitors are being developed for inflammatory disorders (Harris et al., 2017) and these may work only in the subset of patients where the cause is a defect in the early checkpoint. The results from these trials will be interesting as they would provide evidence for whether ripoptocide underlies inflammatory disorders in humans.

**FIGURE 3:**
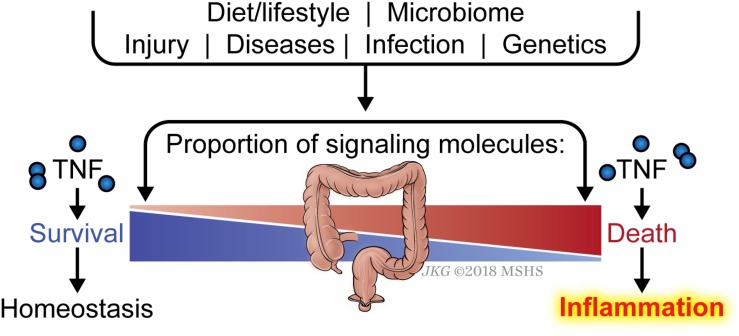
Balance between checkpoint enforcement and disassembly determines sensitivity to ripoptocide-dependent inflammation. Whether a tissue develops sensitivity to TNF-induced ripoptocide and subsequent inflammation may be determined by the level of early checkpoint regulators in the cells within the tissue. For example, if cells from an individual express high level of LUBAC (which enforces checkpoint) but low level of CYLD (which disassembles checkpoint), they would be more resistant to TNF-induced ripoptocide. That individual would be more resistant to ripoptocide-mediated inflammation. Conversely, if cells from an individual express low LUBAC but high level of CYLD expression, that individual would be more prone to ripoptocide and hence inflammation. Individual to individual variation in expression of checkpoint regulators could be due to allelic differences, epigenetic differences and a host of environmental factors extrinsic to the cells. Prior infections or injury could alter expression level via changes in transcription factor activity and chromosomal accessibility. Elements within the diet or byproducts of diet breakdown could directly alter the protein level or functionality of the checkpoint regulators. Encoded gene products or byproducts of metabolism from microbes (commensals or pathogens) may have the same effect. These factors that modulate expression/activity of checkpoint regulators and how they work together to confer sensitivity to ripoptocide are poorly understood.

Another key question is why an individual develops sensitivity to death and therefore inflammation, whereas another individual does not. The list of signaling molecules that constitute the early checkpoint is quite numerous and is likely to grow as we gain more understanding of this checkpoint. Defective expression in one of these genes or more likely, a combination of several genes, would be expected to render the affected cell sensitive to TNF-induced ripoptocide. It is likely that the balance between signaling molecules that enforce the checkpoint (i.e., pro-survival molecules) versus those that disassemble the checkpoint (i.e., pro-death molecules) determine sensitivity to ripoptocide and subsequent inflammation (Figure 3). There is an array of factors that could impact the expression levels of these molecules. Foremost, the genetics of the individual can determine the relative expression of the two opposing classes of molecules in the affected cells. Expression can be further tuned by epigenetic regulation in response to cell extrinsic environmental cues. These cues could come from prior infections, tissue injury, microbiome and diet. In addition to affecting gene expression, microbial-encoded molecules or the products of their metabolism could directly affect the function or availability of these checkpoint molecules via post-translational mechanisms. Cell intrinsic factors and environmental signals likely combine to determine the expression and function of the two opposing classes of molecules. The combinatorial effects from multiple hits may ultimately tip the balance in favor of RIPK1-dependent death and inflammation. Since most human inflammatory disorders are chronic, even a small change in the balance between pro-survival and pro-death molecules in the early checkpoint may lead to disease progression over time without having a significant disruption of homeostasis at any given point.

## Conclusion

The toggling of RIPK1 between its survival-signaling and death-signaling functions, regulated by an early ubiquitin switch, provides a molecular explanation for the long-known capability of TNF to induce either cell survival or death (O’Donnell et al., 2007). An elaborate machinery exists to regulate the non-degradative ubiquitination of RIPK1 as a checkpoint against death. Failure to hold this checkpoint results in ripoptocide. While a number of molecules are now known to regulate this checkpoint, the list is likely to grow. The quest in future studies will be to understand how the different molecules in the checkpoint themselves are regulated. Currently, we have a limited understanding of the genetic, epigenetic and post-translational mechanisms that determine whether the early checkpoint holds or fails. Further insights into these mechanisms will allow us to fully manipulate this checkpoint for therapeutic purposes. Strategies to reinforce the checkpoint and prevent ripoptocide may be clinically beneficial in inflammatory disorders and transplantation. Conversely, disrupting the checkpoint and inducing ripoptocide may be beneficial in cancer, vaccines and infectious diseases.

## Author Contributions

All authors listed have made a substantial, direct and intellectual contribution to the work, and approved it for publication.

## Conflict of Interest Statement

The authors declare that the research was conducted in the absence of any commercial or financial relationships that could be construed as a potential conflict of interest.
